# Data and Safety Monitoring Board Best Practices in Clinical Trials

**DOI:** 10.7759/cureus.107661

**Published:** 2026-04-24

**Authors:** Gerald L Klein, Roger E Morgan, Melissa Palmer, Jamie Lazar, Freddy Byrth, Wayne M Dankner, Jamison Chang, John Hinkle, Jim Sergi

**Affiliations:** 1 Medical Affairs, MedSurgPI LLC, Pittsboro, USA; 2 Hepatology, Liver Consulting, LLC, Dix Hills, USA; 3 Data and Safety Monitoring Board (DSMB) Coordination, Independent Research, Wilmington, USA; 4 Clinical Operations, MedSurgPI LLC, Pittsboro, USA; 5 Pediatrics, DankMD Consulting, Carrboro, USA; 6 Medical Affairs, Grifols, Durham, USA; 7 Medical Affairs, Early Phase Sciences, Inc., Magnolia, USA; 8 Medical Affairs, Qnova LifeSciences, Columbia, USA

**Keywords:** adaptive design, clinical trials, data safety and monitoring, good clinical practice, independent oversight, participant safety, pharmacovigilance, regulatory compliance, risk-based monitoring, trial governance

## Abstract

A Data and Safety Monitoring Board (DSMB), also referred to as a Data Monitoring Committee (DMC), plays a critical role in safeguarding participant safety and ensuring the integrity of clinical trials. This article provides a narrative overview of best practices in DSMB structure, governance, and operations, informed by regulatory guidance from the U.S. Food and Drug Administration (FDA), National Institutes of Health (NIH), European Medicines Agency (EMA), and selected literature, as well as practical experience in clinical trial oversight. Key domains addressed include DSMB composition and independence, charter development and governance, data access and integrity, safety monitoring processes, interim analyses and adaptive decision-making, confidentiality, and sponsor responsibilities. The article also discusses common operational challenges and mitigation strategies, along with emerging trends such as Bayesian monitoring approaches and decentralized trial oversight. Rather than prescribing specific statistical thresholds, the manuscript emphasizes principles for maintaining scientific rigor, ethical oversight, and regulatory alignment. This overview is intended to support sponsors, Clinical Research Organizations (CROs), and DSMB members in implementing effective and compliant safety monitoring practices across a range of clinical trial settings.

## Editorial

Introduction

A Data and Safety Monitoring Board (DSMB), also referred to as a Data Monitoring Committee (DMC), is an independent group of experts external to the sponsor and clinical sites and tasked with the ongoing assessment of safety (sometimes efficacy and/or futility), and trial conduct in ongoing clinical studies [1]. Their unbiased oversight mitigates ethical, scientific, and regulatory risks, especially in multi-centered or double-blinded randomized trials with investigational products and vulnerable populations (e.g., potentially fragile populations, such as children, pregnant women, the very elderly, terminally ill, or of diminished mental capacity) with potential significant risks to the participants or mortality endpoints [2]. Inadequate DSMB governance could lead to missed safety signals, delaying risk mitigation, and noncompliance, potentially compromising participant welfare and trial validity. This article outlines the best practices that sponsors, Clinical Research Organizations (CROs), and DSMB members should follow to uphold participant safety and trial integrity.

Regulatory background

Several regulatory authorities and professional bodies have issued guidance on the operation and use of DSMBs. The International Council for Harmonisation ICH E6(R3) Good Clinical Practice guideline recommends DSMB oversight for studies involving high-risk or those that have mortality as the endpoint. This update also integrates DSMB into a risk-based quality management framework, emphasizing proactive and continuous safety oversight throughout the trial lifecycle [1,3].

Although the Food and Drug Administration (FDA) does not mandate DSMBs for all Phase 1 clinical trials, except those that are known to have a significant risk to participants, we suggest their use as a best practice [4]. As first-in-human studies, these trials may carry the risk of significant or serious adverse events (SAEs) that may be unknown, significant, or not fully established [5]. Examples of this are in oncology and other high-risk disorders where there is a potential for severe toxicity and mortality. Relying on Principal Investigator (PI)-led safety oversight or the Safety Review Committee (SRC) or Safety Monitoring Committee (SMC), often composed of the PI, the medical monitor, and a sponsor-appointed representative, may introduce bias and lack the independence necessary for objective safety evaluation. A well-constituted DSMB can operate at a comparable speed, often through emails, and enable timely decisions without added delays. It can convene ad hoc meetings promptly for urgent safety or efficacy concerns.

The FDA strongly recommends establishing a DMC if trial subjects are at risk of serious morbidity or mortality (e.g., hospitalization, heart attack, stroke, death) [6]. In addition to the effects of the subject’s condition, investigational products may cause serious unexpected adverse events - an important reason to consider monitoring interim results using a DMC. In cases where an assessment of causality can be made based on a single event (e.g., agranulocytosis, Stevens-Johnson syndrome), the sponsor’s internal safety management team or another entity responsible for reviewing safety data may be able to identify a potential risk and bring it to the attention of the sponsor and regulators. In cases where the event may be anticipated to occur in the population enrolled in the trial regardless of the intervention (e.g., myocardial infarctions in an older population) or could be related to other treatments being administered, the relationship between the investigational product and the adverse events will be less clear. In these cases, it is often critical to conduct an analysis of safety data to determine whether, for investigational drugs, there is a reasonable possibility that the adverse event was caused by the investigational drug or whether, for investigational devices, it was caused by or associated with the investigational device [7]. In such cases, a DMC or another independent entity should review aggregate safety reports across study arms [8].

Composition and independence

DSMBs should be composed of experienced experts who are truly independent from the sponsor. Members must have no financial, scientific, or operational conflicts of interest with the investigative sites involved in the study or with the sponsor or investigational product [9]. The coauthors recommend that conflicts of interest be disclosed and updated regularly. While regulatory guidance does not mandate a specific number of physician members, the coauthors further note that governance best practice supports including at least three voting physicians (MDs) on a DSMB to ensure the ability to reach a clear majority and to support clinically informed, ethically sound safety decisions.

DSMBs are charged with safeguarding trial participants through a periodic review of accumulating safety and efficacy data, and with making recommendations regarding the continuation, modification, suspension, or termination of a study [3]. These determinations frequently arise in high-stakes circumstances involving serious adverse events, emerging mortality imbalances, unexpected toxicities, or evolving risk-benefit considerations. In such contexts, a voting structure that permits deadlock introduces unnecessary risk. A DSMB composed of an even number of physicians may reach a tied vote on a safety matter, potentially delaying urgent action. In contrast, a minimum of three physician members establishes an odd-numbered clinical voting core, reducing the probability of procedural impasse and promoting timely, decisive recommendations. The importance of avoiding a deadlock is consistent with broader governance principles applied across regulatory and institutional decision-making bodies [10].

Beyond voting mechanics, the inclusion of three physicians enhances the breadth and depth of clinical expertise available to the DSMB. Clinical trials today, particularly those involving biologics, immunotherapies, gene and cell therapies, device/drug combinations, and adaptive designs, often generate complex safety signals requiring nuanced interpretation [11]. A three-physician structure permits representation of complementary expertise, such as disease-specific knowledge, clinical pharmacology, and prior trial oversight experience. This diversity strengthens the board’s ability to distinguish disease progression from treatment toxicity, to evaluate exposure response relationships in safety signals, and to assess the clinical significance of statistical findings. The FDA’s guidance on DMCs emphasizes that members must possess sufficient clinical expertise to evaluate participant safety and trial integrity [3]. Similarly, International Council for Harmonisation of Technical Requirements for Pharmaceuticals for Human Use (ICH) E6(R2) and the draft ICH E6(R3) underscore risk-proportionate oversight and the need for appropriately qualified experts in trial governance [2]. While these documents do not mandate a minimum number, they implicitly support a composition adequate to address the scientific and safety complexity of the trial at hand. In higher-risk or novel therapeutic studies, a three-physician minimum represents a reasonable baseline.

From an ethical and legal perspective, DSMBs function as an independent protectorate for research participants [11]. Decisions to continue or halt a study must be defensible to sponsors, regulators, institutional review boards, and, if necessary, regulatory agencies. A safety recommendation supported by three independent physician votes demonstrates broader clinical consensus and reduces the perception that a pivotal determination rested on a narrow or potentially divided judgment. In an era of increasing regulatory scrutiny and sponsor awareness of liability exposure, robust medical representation on the DSMB strengthens the credibility of oversight decisions.

The interaction between statistical and clinical interpretation further underscores the need for multiple physicians. Although DSMBs appropriately include a biostatistician, statistical signals, particularly in early-phase or small-sample trials, require careful clinical contextualization [3,11]. A three-physician voting group ensures that statistical thresholds are interpreted within a clinically meaningful framework, preventing overreliance on numerical criteria divorced from real-world patient impact.

The expanding use of DSMBs in earlier-phase and increasingly complex trials supports this structural minimum. National Institutes of Health (NIH) policy and international best practices recognize the growing importance of independent monitoring even in Phase 1 and other high-risk studies [2,9]. As therapeutic modalities evolve, safety signals may be mechanistically novel, delayed, or atypical. A broader physician base enhances the board’s capacity to deliberate thoughtfully and act decisively under uncertainty.

In summary, while no regulation explicitly requires three physician members on a DSMB, principles of governance, risk management, regulatory alignment, and ethical oversight strongly support this minimum. An odd-numbered physician voting core prevents deadlock in safety decisions, broadens clinical expertise, strengthens defensibility, and enhances participant protection. For contemporary clinical trials, particularly those involving novel or higher-risk interventions, a DSMB composed of at least three voting MDs represents sound best practice in clinical trial safety oversight.

The DSMB Chair should be filled by an individual with extensive experience in both DSMB operations and clinical trial conduct. The Chair is responsible for leading deliberations and serving as the primary liaison with the study sponsor and the CRO [11]. Sponsor preferences for the Chair may differ: some may prefer physicians who are board-certified in the study’s therapeutic area, while others prefer those specialized in safety monitoring and clinical trial conduct. Although the selected physicians are not required to be formally trained in the targeted therapeutic area, ideally, they should possess both safety expertise and relevant clinical trial experience in the therapeutic area or in the study indication. A statistician with expertise in DSMB operations is essential for many clinical trials, particularly those that include interim analyses to determine whether a study should continue as planned or be stopped early for futility [10]. Familiarity with the therapeutic area can also be valuable, at times, if it helps ensure that interim data are interpreted within the appropriate clinical context.

Charter and governance

A comprehensive DSMB Charter governs operations and ensures consistency with the protocol. The charter should clearly outline the roles and responsibilities of the DSMB, sponsor, and CRO. The charter also outlines the frequency, format, and procedures for meetings; data review protocols; statistical methods (e.g., alpha spending functions) and decision-making processes, including voting procedures and criteria for trial modification, pausing, or termination [6]. The charter should also define rules for data access control for sensitive unblinded data, and establish clear communication pathways with the CRO, sponsor, and regulatory authorities. Additionally, the charter must address succession planning and procedures for member replacement. Provisions for data access should be explicitly stated, including the types of data to be reviewed, the timing of data cutoffs, and the handling of revised or updated information. These expectations should also be incorporated in the DSMB-sponsor-CRO agreements.

Data access and statistical reporting

DSMBs require timely access to accurate, relevant data, whether appropriately blinded or unblinded, to enable informed decision-making. Independent statisticians, separate from the sponsor, should prepare both open-session reports (blinded to preserve integrity) and closed-session reports (unblinded). These reports may include enrollment trends, tolerability assessments, summaries of adverse events (AEs) and SAEs, efficacy endpoints, and any interim analyses aligned with predefined statistical stopping boundaries [12].

The independent DSMB statistician should develop a dedicated DSMB Statistical Analysis Plan (SAP) that details methods for multiplicity adjustments, sensitivity analyses, and estimand definitions per ICH E9(R1). This statistician will also collaborate with the sponsor’s or CRO’s data management team to create a Data Management Plan (DMP). The DMP should specify the data to be transferred, formatting standards (e.g., Clinical Data Interchange Standards Consortium (CDISC)), validation procedures, and delivery timelines (e.g., within seven days post-data cutoff) [13]. All data provided to DSMB members must be timely, usable, and aligned with the board’s review schedule and decision-making requirements.

Safety monitoring best practices

DSMBs should conduct systematic reviews of cumulative safety data throughout the trial, including but not limited to adverse events, serious adverse events, deaths, adverse events of special interest, laboratory abnormalities, and withdrawals due to adverse events [6,11]. These reviews should incorporate structured assessments of causality using standardized methods when available. They should also include detailed adverse event narratives and exposure-adjusted event rates (e.g., events per 100 patient-years) to support informed risk-benefit evaluations as applicable. In addition to their core safety oversight responsibilities, DSMBs should monitor key operational and trial-integrity metrics. These include enrollment pace, which directly affects endpoint accrual and overall trial feasibility. DSMBs should also assess the occurrence of major protocol violations, as these can compromise data integrity and the interpretability of trial outcomes.

Interim analyses and trial modifications

Interim analyses, when undertaken, must be prospectively specified in the study protocol and statistical analysis plan to preserve trial integrity and control the risk of operational bias. The DSMB should be explicitly empowered to make recommendations based on interim findings [14]. This should include continuation of the trial as planned, implementation of protocol modifications, such as sample size adjustments or endpoint refinements, temporary pauses in enrollment to allow further evaluation, or early termination of the study due to futility, overwhelming efficacy, or safety concerns. In trials employing adaptive designs, additional safeguards should be implemented. For example, the use of independent statistical centers, to prevent unintentional unblinding and to maintain the integrity of blinding throughout the conduct of the trial [15].

Confidentiality and blinding

Strict confidentiality and maintenance of blinding are essential to preserve trial integrity and prevent bias in DSMB operations. To support these principles, DSMB meetings typically follow a three-part structure consisting of open and closed sessions [16]. Open sessions are attended by DSMB members, along with the sponsor and CRO representatives. They focus on the review of blinded data, operational updates, such as enrollment status and site performance, and logistical matters, without disclosure of treatment assignments. Closed sessions are restricted to DSMB members, the independent statistician, and, when necessary, an unblinded administrator. They are used to review unblinded safety and efficacy data (as applicable), deliberate on sensitive issues, and formulate formal recommendations. These recommendations may include continuation of the trial as planned [17], proposed protocol modifications, such as dose adjustments or changes to inclusion criteria, or advice to pause enrollment or terminate the study early due to safety concerns, futility, or overwhelming efficacy. In addition, the DSMB may provide blinded operational feedback to the sponsor in addition to a recommendation section to improve trial conduct while maintaining confidentiality and preserving the integrity of the study.

Meeting logistics and documentation

DSMBs should convene at regular intervals, typically every three to six months, with more frequent meetings for higher-risk studies or when predefined milestones are met (e.g., a predefined percentage of participants complete a set duration on study, as outlined in the protocol or charter) [18].

Meeting frequency and scheduling should be driven by predefined thresholds and emerging trial needs. Ad hoc DSMB meetings should be convened promptly in response to emerging safety concerns, unexpected adverse events, or urgent efficacy signals. In early-phase studies, such as Phase 1 dose-escalation trials, expedited reviews are particularly important to evaluate accumulating safety data and to inform decisions regarding dose progression, modification, or stopping. To support effective deliberation, all relevant meeting materials, including safety reports and data summaries, should be distributed to DSMB members approximately 5 to 10 days in advance to allow adequate preparation. Each meeting should be thoroughly documented in formal minutes that capture attendance, data reviewed, decisions made, the rationale for those decisions, and any dissenting opinions, thereby supporting transparency, auditability, and regulatory compliance [19].

Sponsor responsibilities

Sponsors bear primary responsibility for supporting DSMB operations while preserving the board’s independence to ensure unbiased oversight. This responsibility includes providing clean, accurate, and timely data through validated systems, with predefined data cutoffs and formats aligned with the DSMB charter and data management plan, as well as offering appropriate statistical support through independent experts for the preparation of blinded and unblinded reports and logistical resources such as secure platforms for data sharing and meeting facilitation. At the same time, sponsors must respect DSMB independence by avoiding any attempts to influence deliberations, prematurely access unblinded data, or override DSMB recommendations. Any sponsor resistance, interference, or undue influence can undermine DSMB validity, leading to biased outcomes, regulatory noncompliance, delayed risk mitigation, and compromised participant safety [19].

Challenges and mitigations

DSMB operations are subject to several common challenges that, if not proactively addressed, can compromise participant safety, trial integrity, and regulatory compliance. These challenges include the presence of conflicted members, where financial, scientific, or operational conflicts of interest are not adequately identified or managed. Potential biasing deliberations underscore the need for annual disclosures and independent vetting in line with FDA guidance. Ambiguous DSMB charters that lack clearly defined roles, responsibilities, or decision criteria can lead to inconsistent oversight. This should be avoided by developing charters with explicit provisions for meeting frequency, data review procedures, and escalation pathways aligned with the study protocol. Delayed or incomplete access to data can hinder timely, informed decision-making and increase the risk of missing emerging safety signals, highlighting the importance of strict data management timelines to ensure prompt data transfer and analysis [18]. Sponsor resistance or attempts to influence DSMB deliberations undermine board independence and should be mitigated through clearly defined boundaries in charters and contractual agreements. Inadequate documentation, including incomplete meeting minutes or insufficient records of deliberations, can raise concerns during audits and inspections; therefore, comprehensive documentation of attendance, data reviewed, decisions made, and dissenting opinions is essential to support transparency and regulatory adherence. Additionally, failure to convene ad hoc DSMB meetings when warranted to evaluate emerging or serious safety concerns represents a critical operational gap. Finally, unclear or poorly specified interim analysis plans may inflate error rates or lead to unintended unblinding; prespecifying interim analysis methods, including stopping boundaries, is necessary to preserve statistical validity and overall trial integrity [20].

Future directions

Emerging innovations in DSMB operations offer opportunities to enhance efficiency, inclusivity, and adaptability in clinical trials. The increased use of Bayesian monitoring allows flexible interim analyses and adaptive decision-making [21]. This is achieved by incorporating prior information and accumulating data in real time to optimize trial efficiency and safety oversight. DSMB processes are also evolving to support decentralized clinical trials, with tailored approaches such as remote data review, virtual meetings, and hybrid monitoring models that accommodate decentralized elements while maintaining rigorous safety evaluations [12]. In parallel, there is growing emphasis on increasing diversity in DSMB composition, including diversity of expertise, demographics, and perspectives, to strengthen equitable risk assessment and reduce bias in data interpretation. Advances in artificial intelligence offer additional support through enhanced data pattern recognition and early signal detection; however, human judgment must remain central to final DSMB deliberations to ensure ethical, scientific, and regulatory rigor. Finally, for resource-constrained sponsors, program-level DSMBs spanning multiple studies within a development program may provide a cost-effective and consistent oversight model, provided that charters and agreements clearly empower the board to adjust monitoring intensity based on individual study risk profiles.

Conclusion

DSMBs are essential for protecting participant safety, upholding scientific rigor, and achieving regulatory compliance in clinical trials. Adhering to the best practices detailed in this article, which include multidisciplinary composition, clear governance, systematic data reviews, safety monitoring, and adaptive decision-making, enables sponsors, CROs, and DSMB members to conduct ethical, efficient, and successful trials. This approach supports the advancement of innovative therapies while effectively mitigating risks.
